# Femtosecond time-resolved photodissociation dynamics of methyl halide molecules on ultrathin gold films

**DOI:** 10.3762/bjnano.2.65

**Published:** 2011-09-20

**Authors:** Mihai E Vaida, Robert Tchitnga, Thorsten M Bernhardt

**Affiliations:** 1 Institute of Surface Chemistry and Catalysis, University of Ulm, Albert-Einstein-Allee 47, 89069 Ulm, Germany

**Keywords:** femtosecond laser spectroscopy, gold, methyl halide photodissociation, surface chemistry, time-of-flight mass spectrometry

## Abstract

The photodissociation of small organic molecules, namely methyl iodide, methyl bromide, and methyl chloride, adsorbed on a metal surface was investigated in real time by means of femtosecond-laser pump–probe mass spectrometry. A weakly interacting gold surface was employed as substrate because the intact adsorption of the methyl halide molecules was desired prior to photoexcitation. The gold surface was prepared as an ultrathin film on Mo(100). The molecular adsorption behavior was characterized by coverage dependent temperature programmed desorption spectroscopy. Submonolayer preparations were irradiated with UV light of 266 nm wavelength and the subsequently emerging methyl fragments were probed by photoionization and mass spectrometric detection. A strong dependence of the excitation mechanism and the light-induced dynamics on the type of molecule was observed. Possible photoexcitation mechanisms included direct photoexcitation to the dissociative A-band of the methyl halide molecules as well as the attachment of surface-emitted electrons with transient negative ion formation and subsequent molecular fragmentation. Both reaction pathways were energetically possible in the case of methyl iodide, yet, no methyl fragments were observed. As a likely explanation, the rapid quenching of the excited states prior to fragmentation is proposed. This quenching mechanism could be prevented by modification of the gold surface through pre-adsorption of iodine atoms. In contrast, the A-band of methyl bromide was not energetically directly accessible through 266 nm excitation. Nevertheless, the one-photon-induced dissociation was observed in the case of methyl bromide. This was interpreted as being due to a considerable energetic down-shift of the electronic A-band states of methyl bromide by about 1.5 eV through interaction with the gold substrate. Finally, for methyl chloride no photofragmentation could be detected at all.

## Introduction

The understanding of the mechanisms involved in the light-induced excitation and fragmentation of organic molecules on metal substrates is of great importance in several research areas and applications connected to surface chemistry and catalysis. Photostability, photooxidation, and photocatalysis are important concepts in this respect that attract considerable interest in the fields of nanotechnology and surface engineering [1–2]. The present investigation focuses on fundamental mechanistic aspects associated with the interaction of small organic molecules with metal surfaces. For this purpose, ultrafast, time-resolved laser spectroscopy was applied to provide insight into light-induced molecular fragmentation on surfaces on the time-scale of nuclear motion. The system under investigation was a nanoscale organic–inorganic layer structure composed of an organic overlayer adsorbed on a weakly interacting ultrathin gold film on a Mo(100) single crystal substrate. Methyl halide molecules are simple pseudo-diatomic photochemical model systems, which have been studied in great detail in the gas phase (see, e.g., [3–7]) as well on several metallic and nonmetallic solid substrates (see, e.g., [8–14]). The adsorption of methyl iodide on a gold surface has been previously investigated [9,11]. However, nothing has been reported so far about the adsorption of methyl bromide or methyl chloride on gold. Also, no ultrafast time-resolved laser investigations of methyl halide molecules on metal substrates have been performed so far. Only recently, the femtosecond (fs)-laser time-resolved photodissociation of methyl iodide and methyl bromide on oxide-supported gold nanoparticles was investigated [15–17]. The gold films employed in the presented experiment for the investigation of photoinduced reaction dynamics of methyl halide molecules on metallic supports were grown on Mo(100).

The central questions of the present investigation are concerned with the dependence of the photoexcitation and the subsequent photodissociation dynamics on the electronic structure of the organic adsorbates and, even more importantly, on the interaction with the substrate surface. Furthermore, the obtained results provide insight into fundamental issues of the ultrafast reaction dynamics of molecules on metallic surfaces and the influence of organic-molecule–inorganic-substrate interactions on the molecular photoreaction dynamics.

## Results and Discussion

In the following, the results of the molecular adsorption studies and the investigations of the photodissociation dynamics are presented for the three molecular-adsorbate systems investigated. The characterization of the ultrathin gold films on Mo(100) that have been employed as a substrate in the present study is detailed in Supporting Information File 1.

### Adsorption of methyl halide molecules on Au/Mo(100)

#### Methyl iodide

**Figure 1:**
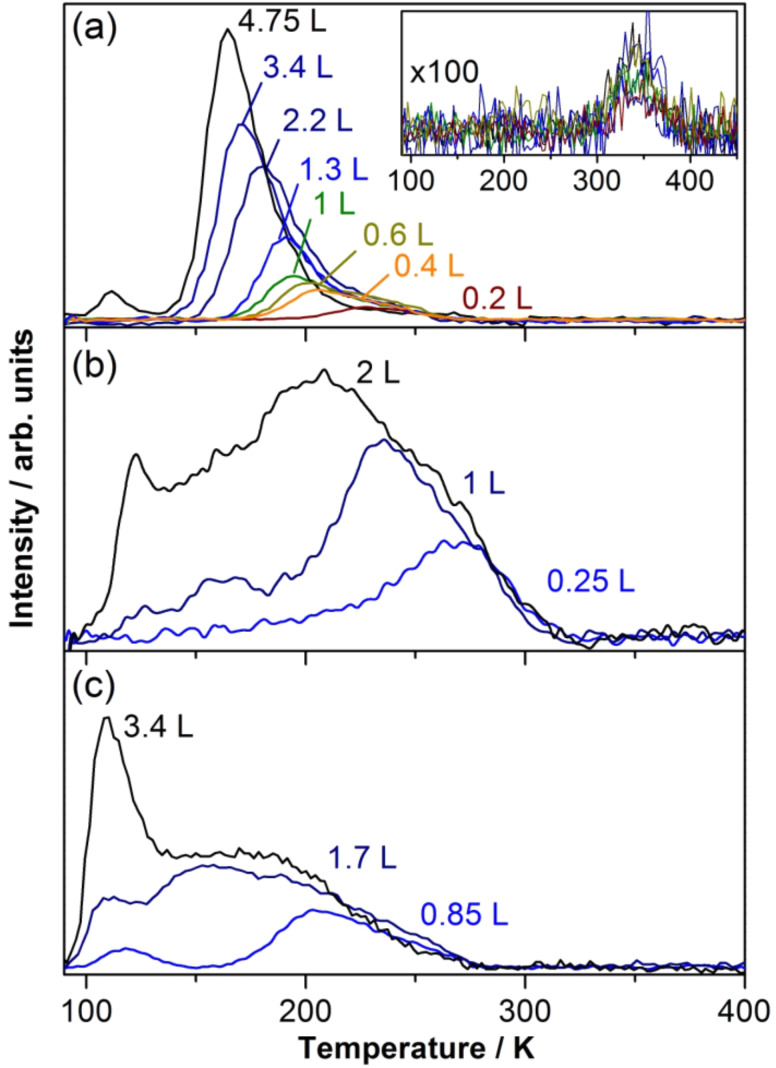
Temperature-programmed desorption/reaction spectra showing the molecular desorption of: (a) CD_3_I, (b) CH_3_Br and (c) CH_3_Cl from a 10 ML Au film on Mo(100). The numbers indicated next to the spectra represent the methyl halide exposure in units of Langmuir. The inset displays the C_2_D_6_ signal evolution. The C_2_D_6_ signal intensity has been multiplied by a factor of 100. All spectra were obtained with a heating rate of 2 K/s.

Figure 1a shows temperature-programmed desorption (TPD) spectra recorded after dosing different amounts of CD_3_I molecules at 90 K onto a 10 ML gold film grown on Mo(100). Similar to the TPD investigations of methyl iodide on Au(100) [11] and on Au(111) [9] surfaces that have been reported in the literature, we found that most of the first layer desorbs without fragmentation. At submonolayer coverage just one desorption peak, which shifts to lower temperature with increasing coverage, appears in the TPD spectra. A similar desorption feature has been observed for CH_3_I [10,18] and CH_3_Br [12] on MgO, CH_3_Br on LiF [19], and CH_3_Cl on Pd(100) [20], as well as for CH_3_Cl, CH_3_Br and CH_3_I on GaAs(110) [21–22], and the coverage dependence is attributed to the adsorbate–adsorbate repulsion that results from the interaction between the static dipole moments of adsorbed molecules. Due to this lateral repulsion between the adsorbate molecules, the activation energy for desorption decreases with increasing coverage and, hence, the desorption temperature decreases. The completion of the first monolayer of CD_3_I molecules appears for doses just below 4.75 L, in agreement with the previous investigations on Au(100) [11]. The peak below 140 K in Figure 1a is due to the multilayer desorption. Higher coverages were not investigated in this experiment.

About 4% of the CD_3_I molecules of the first layer dissociate and produce ethane. The inset in Figure 1a shows the evolution of the ethane signal in the temperature-programmed reaction of CD_3_I molecules on the 10 ML Au film on Mo(100). The ethane signal starts just below 300 K, extends up to 400 K and presents a maximum at 350 K. Based on similar results for Au(100) [11] and Au(111) [9], the ethane formation is thought to be a consequence of methyl iodide dissociation followed by coupling between the methyl radicals. Since the temperature at which ethane appears is higher than that for the ethane molecular desorption (below 100 K), it was concluded that the ethane formation is the rate-determining step as opposed to the desorption [11]. Furthermore, it can be assumed that the methyl iodide molecules involved in the thermal reaction are adsorbed on defect sites and dissociate close to the ethane-appearance temperature (300 K) [11]. The remaining iodine atoms resulting from dissociation should desorb from the surface as atoms at temperatures higher than 650 K [23] (not investigated here). The intensity of the ethane signal saturates at a CD_3_I coverage corresponding to an exposure of about 0.75 L in agreement with the previous investigation of Yang et al. [11]. No other thermal reaction products were detected.

#### Methyl bromide

The adsorption behavior of methyl bromide on a gold surface was also investigated by means of TPD spectroscopy, and the results are displayed in Figure 1b. Methyl bromide generally desorbed at higher temperatures from the gold substrate as compared to methyl iodide. For an exposure of 0.25 L the CH_3_Br desorption began at around 150 K and extended to 310 K, with an intensity maximum at about 275 K. For an exposure of 1 L CH_3_Br, a decrease of the maximum desorption temperature to about 235 K was observed. Additionally, a new desorption feature appeared below 200 K. When 2 L of CH_3_Br were dosed onto the Au film, a distinct desorption peak at 120 K was observed separate from the main desorption peak as observed at lower coverages, with the maximum now shifted to 205 K. No investigations of the adsorption of methyl bromide molecules on a gold surface have been reported so far in the literature. The interpretation of the features displayed in Figure 1b is therefore based on TPD experiments with methyl halide molecules adsorbed on different substrates, which have been reported in the literature (see above). The desorption peak above 170 K is assigned to the molecular desorption of CH_3_Br molecules from the gold surface. Its shift to lower temperature with increasing adsorbate coverage is attributed also to a repulsive desorption that is caused by the interaction between the static dipole moments of the adsorbed molecules. A similar desorption characteristic was observed for submonolayer coverages of CH_3_Br on Ru(001) [13]. The distinct peak at 120 K is assigned to the onset of the CH_3_Br multilayer desorption, which was observed to start at an exposure of 3.4 L on Ru(001) [13]. In contrast to methyl iodide adsorbed on a gold surface, no thermally induced reaction products of CH_3_Br, such as methane or ethane, were detected. As well, no evidence for methyl bromide dissociation subsequent to adsorption on the Au substrate was found.

#### Methyl chloride

Similar to the case of methyl bromide, no desorption studies of methyl chloride on a gold surface have been reported so far. Our results are depicted in Figure 1c. Already at 0.85 L exposure two desorption features were observed. The high-temperature peak shifts to lower temperatures with increasing coverage, indicating again repulsive adsorbate–adsorbate interactions. Interestingly, already at 0.85 L exposure a low-temperature desorption peak also emerges at around 110 K. This peak might indicate early multilayer desorption, which would be in accordance with similar results of CH_3_Cl adsorbed on Ru(001) [13]. In this case the authors reported the onset of multilayer desorption for a methyl chloride exposure of 1.6 L. Also for methyl chloride no thermally induced reaction products were detected.

### Molecular photodissociation dynamics

Molecular photoexcitation on solid surfaces is generally believed to proceed through one of the two following mechanisms:

1. Electron attachment leading to transient negative molecular ions, which in the case of the methyl halide molecules are unstable and subsequently decompose, or
2. direct photoexcitation of the adsorbed molecules to electronically excited states, which will determine the following photoreaction dynamics. For the methyl halide molecules the first optically accessible states belong to the dissociative A-band.

**Figure 2:**
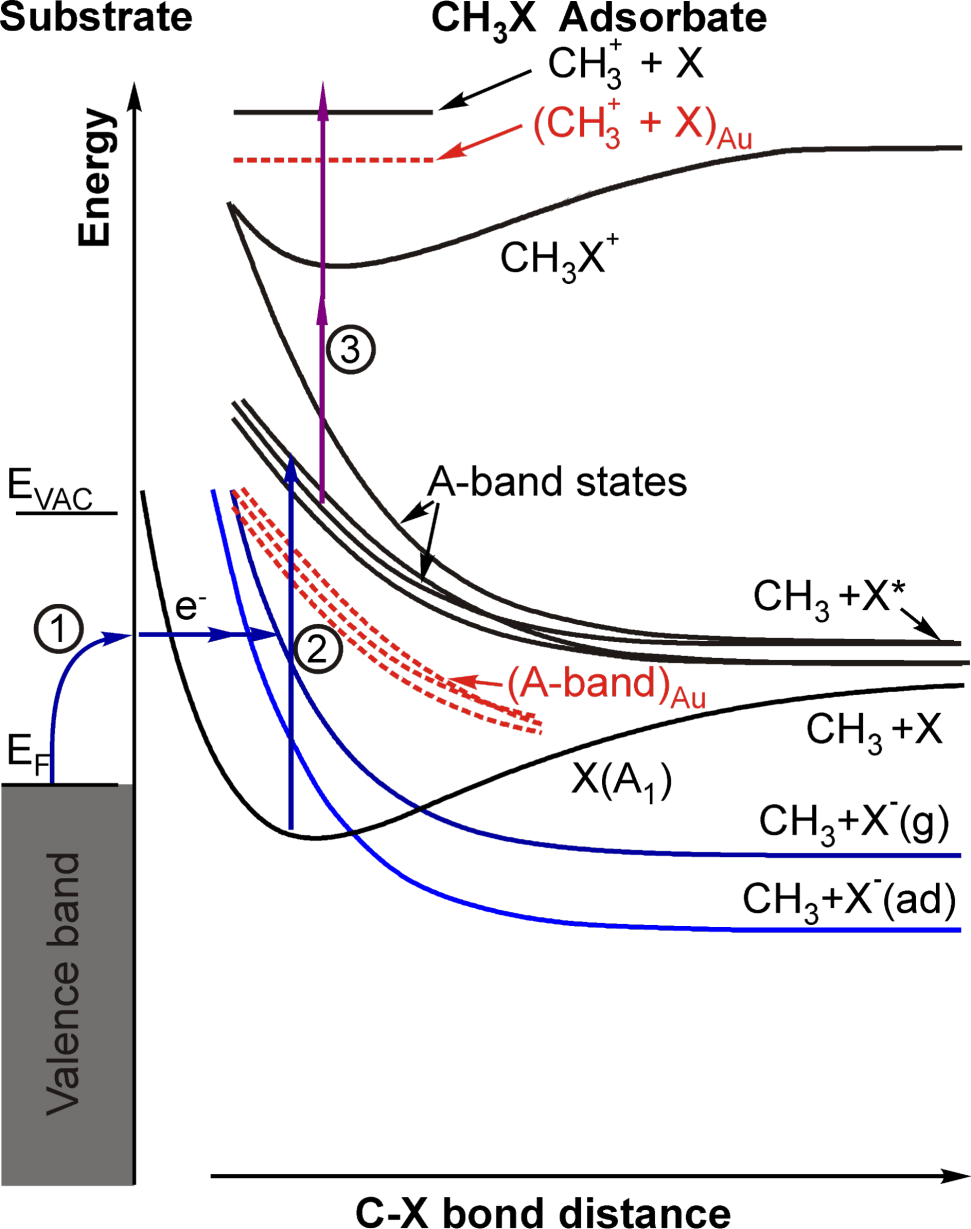
Schematic potential energy diagram for CH_3_X (X = I, Br, Cl) molecules adsorbed on a metal surface. The shape of the potentials and the general idea have been adapted from data on CH_3_Br in [7,24–27]. The numbers of the excitation and detection mechanisms refer to the discussion in the text. The electronic levels that are proposed to be involved in the excitation and detection mechanism on the gold surface are indicated by the dashed red lines.

These two principally possible photoexcitation scenarios are schematically illustrated in Figure 2 for the methyl halide molecules adsorbed on a metal surface. For methyl iodide both reaction pathways are energetically possible with the absorption of one pump-laser photon of 266 nm wavelength. For methyl bromide and methyl chloride the A-band absorption maximum is 6.2 and 7.3 eV, respectively, above the electronic ground state of the free molecules [3] and thus not accessible through one-photon excitation at 266 nm (4.7 eV).

#### Methyl iodide

*Methyl iodide photodissociation on gold:* CD_3_I was adsorbed at submonolayer coverage on a 10 ML Au film on Mo(100) and irradiated with fs-laser pulses of 266 nm wavelength (pump beam, 80 fs, p-polarized, 1–2 mW/cm^2^). For the subsequent fragment ionization a probe laser beam with a wavelength of 333 nm (80 fs, p-polarized, 600 mW/cm^2^) was employed. However, neither photodissociation products nor the parent molecule were detected, independent on the pump–probe delay time. Similar results were reported by White and coworkers for methyl iodide adsorbed on Pt(111) [28–31]. In their work, methyl iodide molecules that were adsorbed on the metal surface were exposed to the full spectrum of an Hg arc lamp. The photodissociation and the desorption of the molecules from the surface was subsequently monitored by means of X-ray photoemission spectroscopy. White and coworkers reported that only 10% of the first CH_3_I layer adsorbed on the Pt(111) substrate dissociated when the sample was irradiated for 120 min with a 100 W Hg arc lamp [29]. In contrast, a facile cleavage of the second layer was observed. According to vibrational spectroscopy investigations, the methyl iodide molecules adsorb on a Pt(111) surface with the halogen atom bound to the surface and with the C–I axis considerably tilted away from the surface normal [32]. On Au(100) an adsorption geometry with the C–I axis almost parallel to the surface plane is even expected for CH_3_I at submonolayer coverages [11]. Under these circumstances, the ground state of the CH_3_I molecule is only slightly perturbed from the gas phase. In contrast, the excited state is strongly bound to the metal surface, which facilitates the recapturing of the ground state before the C–I bond stretches considerably. As a consequence, the molecule will almost instantaneously relax back to the ground state after excitation, and the molecular dissociation will be quenched [29]. Also on an Ag(111) surface the methyl iodide molecules adsorbed at monolayer coverage are photodissociated at a slower rate compared to those adsorbed at multilayer coverages, which also indicates a substantial quenching of the photodissociation when the molecules are in contact with the metal substrate [33–34]. In contrast to CH_3_I, however, the photodissociation of the first monolayer of CH_3_Br and CH_3_Cl is easily promoted on the Pt(111) surface through UV irradiation [24,31,35–36].

**Figure 3:**
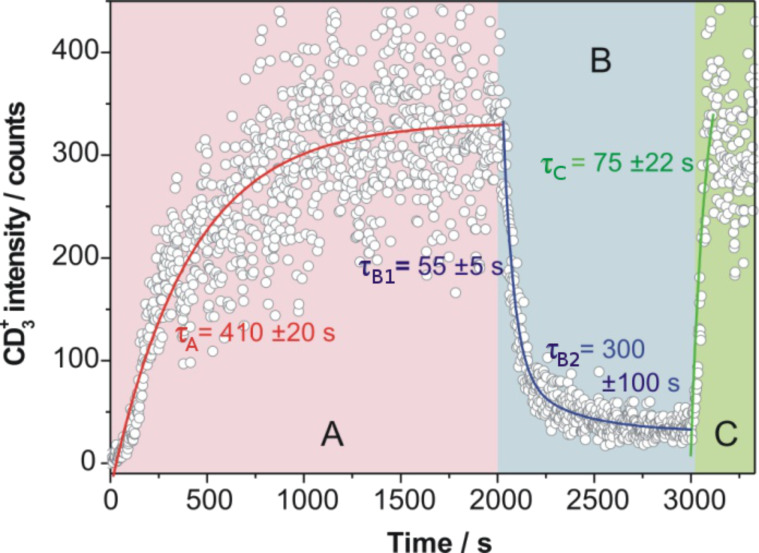
Time evolution of the integral CD_3_^+^ intensity (open circles) during the continuous admission of CD_3_I to a 10 ML Au film on Mo(100). The surface temperature was 120 K. At 2000 s the gas admission was stopped for 1000 s. The solid lines represent the best fits to the different parts of the measured data (A, B and C) with exponential growth and decay functions, as appropriate (see text for more details). Pump pulse: 266 nm, 2 mW/cm²; probe pulse: 333 nm, 600 mW/cm²; pump–probe delay time: 130 fs.

*Trapping of iodine atoms at the surface subsequent to CD_3_I photodissociation on gold:* Figure 3 displays the time evolution of the CD_3_^+^ signal during continuous admission of CD_3_I to a 10 ML Au film on Mo(100). The surface temperature was held at 120 K to ensure a maximum CD_3_I coverage of just about one monolayer (Figure 1a and [11]). In the beginning (period A in Figure 3), the surface was continuously irradiated by the pump- and probe-laser beams. An increase of the CD_3_^+^ signal intensity was observed, starting after a few tens of seconds. The saturation of the CD_3_^+^ signal appeared close to 2000 s. The CD_3_^+^ signal up to 2000 s approximates to an exponential rise function. The obtained time constant is τ_A_ = 410 ± 20 s. After 2000 s (period B in Figure 3), the CD_3_I admission was stopped, but the surface was further irradiated by the laser beams. The CD_3_^+^ signal immediately started to decrease. This decay is best fitted by a second-order exponential decay function. The time constants of the fast and slow decaying parts are τ_B1_ = 55 ± 5 s and τ_B2_ = 300 ± 100 s, respectively. After 3000 s the CD_3_I gas admission was restarted (period C in Figure 3) and the CD_3_^+^ signal exhibited an abrupt increase. The best fit to this signal with an exponential rise function gives a time constant of τ_C_ = 75 ± 22 s.

The initial rise of the CD_3_^+^ signal in Figure 3 (period A) cannot be attributed to the continuous accumulation of CD_3_I molecules onto the surface, since the saturation of the monolayer is expected within less than 10 s. Moreover, a subsequent irradiation of a new (but already covered with CD_3_I) surface area, of the same sample, gave a similar exponential rise of the CD_3_^+^ signal. Therefore, it must be assumed that the result shown in Figure 3 indicates a change in the nature of the surface during laser irradiation. As discussed in the previous section, just a small amount of C–I bond cleavage of CH_3_I at monolayer coverage on Pt(111) was observed after a long period (120 min) of irradiation with a 100 W Hg arc lamp [29]. In the present experiment a much more intense light source, i.e., a fs-laser, was employed. Therefore, in our experiment the dissociation of the CD_3_I adsorbed at monolayer coverage on Au can be assumed to occur at a somewhat faster rate (on the time scale of tens of seconds in contrast to tens of minutes). According to the adsorption geometry and binding energy of methyl iodide molecules on the Au surface [11], we expect the trapping of the iodine atoms at the surface subsequent to CD_3_I photodissociation. Consequently, during the laser irradiation of the sample an iodine film (or a gold–iodine layer) might be formed between the Au surface and the subsequently adsorbed CD_3_I molecules. On this iodine film, the photodissociation of the CD_3_I molecules should not be quenched anymore. The suggestion of the formation of an iodine layer, or at least an iodine containing gold layer, in the present experiment is supported by the investigations of White and coworkers, who observed that the CH_3_I photodissociation on a Pt(111) surface precovered with a monolayer of iodine atoms was not quenched either [37].

The rise time of the CD_3_^+^ signal in Figure 3 (period A) should be related to the formation of the iodine layer. Once the sample is modified, i.e., the light-induced transformation CD_3_I/10 ML Au/Mo(100) → CD_3_I/I/10 ML Au/Mo(100) is completed, the laser can induce the dissociation and fragment desorption of an significant fraction of the CH_3_I molecules that are adsorbed on the iodine layer. The dissociated and desorbed amount of CD_3_I is immediately restored owing to the continuous admission, and a stable methyl desorption signal is established at the end of period A in Figure 3. In period B of Figure 3, the fast decaying part is attributed to the dissociation of the uppermost CD_3_I molecules that were weakly adsorbed on the iodine layer. The slowly decaying part in period B could be attributed to the dissociation of strongly bound CD_3_I molecules, which were possibly surrounded by iodine atoms. Towards the end of period B in Figure 3 it can be expected that the sample surface consisted mostly of iodine atoms adsorbed on the gold film on Mo(100). The considerably faster rise of the methyl signal in period C of Figure 3, when the gas admission was restarted, compared to the one obtained in the beginning of the experiment (see period A in Figure 3), supports the assumption that a change of the surface composition after laser irradiation during continuous methyl iodide admission had occurred. The CD_3_^+^ signal in period C is attributed to the dissociation of CD_3_I molecules adsorbed on the new iodide film that was formed during the previous laser irradiation. The fast rise of the CD_3_^+^ signal then reflects the time needed to saturate this iodine film surface with CD_3_I molecules. The maximum CD_3_^+^ signal intensities reached in both periods B and C coincide. The exact values of the time constants presented in Figure 3 depend of course strongly on the laser intensity. In particular, the rise time in period A can be considerably extended at reduced laser intensity.

**Figure 4:**
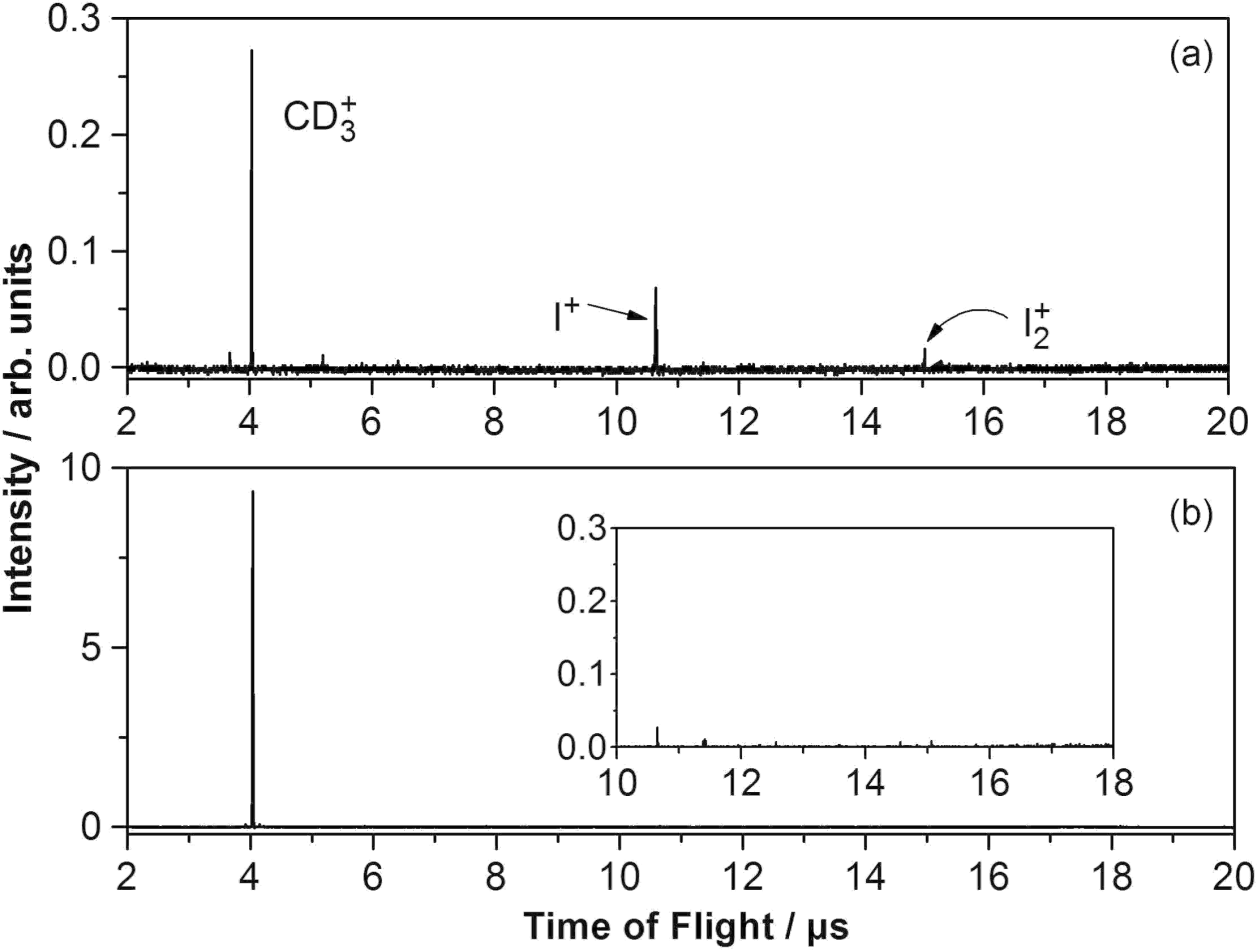
Mass spectra recorded at (a) 150 K and (b) 100 K from the same sample surface as in Figure 3 period C, after 3500 s. The inset presents a zoom-in to the mass spectrum (b). During the recording of the mass spectra, CD_3_I was continuously dosed onto the surface. Pump pulse: 266 nm, 2 mW/cm²; probe pulse: 333 nm, 600 mW/cm²; pump–probe delay time: 130 fs.

In Figure 4 laser desorption mass spectra recorded from the sample surface employed in the previous experiment (Figure 3) after 3500 s are shown. At a surface temperature of 150 K (Figure 4a), signals corresponding to atomic and molecular iodine are detected separate from the methyl mass peak. At 150 K the CD_3_I coverage is below one monolayer (Figure 1a) and therefore, iodine atoms and molecules can also be desorbed by laser irradiation from the iodine layer on the gold surface, which also supports the assumption that iodine atom fragments have been previously trapped on the gold surface.

**Figure 5:**
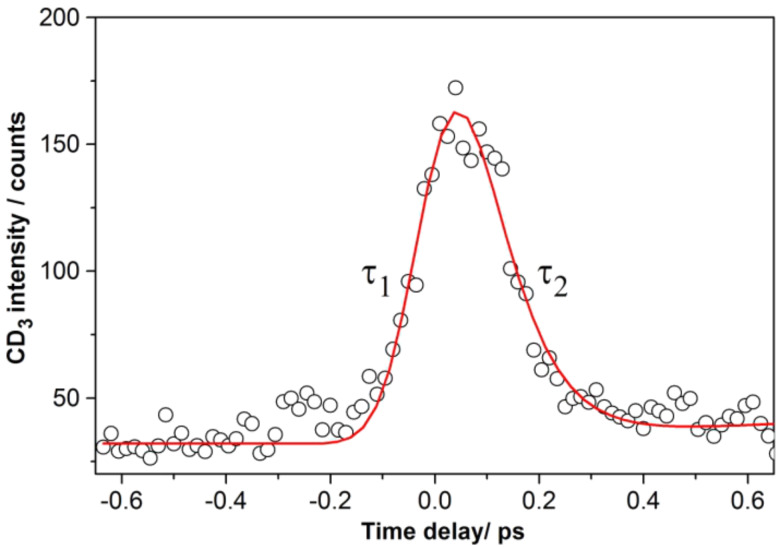
Femtosecond photodissociation reaction of multilayer methyl iodide adsorbed on 10 ML Au/Mo(100) recorded by monitoring the transient methyl cation signal intensity as a function of the pump–probe delay time (pump: 266 nm, 2 mW/cm² and probe: 333 nm 60 mW/cm²). The solid line is the best fit of an exponential “rise and decay” model to the experimental data, yielding the indicated time constants τ_1_ and τ_2_.

In contrast, at 100 K surface temperature multilayer coverages of CD_3_I are possible. The mass spectrum in Figure 4b was obtained at this surface temperature and confirms that, under these multilayer conditions, the atomic and molecular iodine desorption from the iodine layer on the gold surface is suppressed because the iodine layer is completely covered by CD_3_I. In addition, when the sample was held at 100 K the methyl peak intensity was about 35 times higher than that at 150 K owing to the CD_3_I multilayer formation (note that at the chosen delay time of 130 fs no iodine atoms or I_2_ molecules resulting from molecular CH_3_I photodissociation were detected with the employed probe laser beam [38]; the detected I-atoms and I_2_ molecules in the mass spectrum must therefore originate from the iodine layer on the gold surface). On the modified gold surface (I/Au/Mo(100)) it was then possible to record the fs photodissociation dynamics of CD_3_I by monitoring the transient methyl cation signal intensity as a function of the pump–probe delay. The result is shown in Figure 5. The CD_3_^+^ transient signal consists of a peak structure with a maximum at 50 fs. The interpretation of this transient is based on earlier experiments with methyl iodide on an insulating magnesia surface and on the power dependences of the pump and the probe laser, which support a single-photon excitation followed by a two-photon ionization [18,38–39].

Dissociative electron attachment according to excitation mechanism (1) in Figure 2 might be possible. In this way transient CH_3_I^−^ ions would be generated, which would subsequently decompose. However, the formation of methyl cations would in this case involve a transition from the anion, via the neutral, to the cation. Of course, the exact locations of the respective potential energy surfaces are not known, but owing to the steep slope of the potentials at short distances (Figure 2) it is likely that this would require a three-photon transition, which is not in accordance with our observed two-photon ionization probe process. Therefore, it is assumed instead that one single photon of 266 nm excited the adsorbed methyl iodide molecule to the A-band (excitation mechanism (2) in Figure 2). The peak structure is then attributed to the dynamics of the dissociating excited transition state of methyl iodide in the A-band, which can be directly ionized with the highest cross section after 50 fs by two photons of the probe pulse at 333 nm wavelength (excitation mechanism (3) in Figure 2). Subsequent rapid decomposition of the excited methyl iodide cation is proposed to lead to the observed methyl fragment signal. Fitting the experimental data with an exponential “rise and decay” model (convoluted with the laser cross correlation function) yielded the time constants of τ_1_ = 60 ± 10 fs and τ_2_ = 70 ± 10 fs for the rise and decay of the peak structure, respectively.

#### Methyl bromide

**Figure 6:**
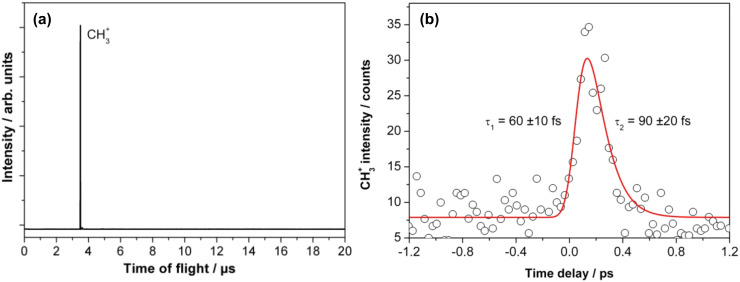
(a) Time-of-flight mass spectrum obtained from 0.25 ML methyl bromide adsorbed on a 10 ML Au film on Mo(100) at 140 fs pump–probe delay time. (b) Transient fs-photodissociation dynamics of CH_3_Br molecules adsorbed on Au(100)/Mo(100) recorded by monitoring the CH_3_^+^ signal as a function of the pump–probe time delay. The surface temperature was 150 K. The solid line is the best fit of an exponential “rise and decay”-model to the experimental data, yielding the indicated time constants τ_1_ and τ_2_. For the shown transient, four single transients were averaged to improve the signal-to-noise ratio. The transient was obtained with 2 mW/cm² pump (266 nm) and 200 mW/cm² probe (333 nm) laser power.

In contrast to methyl iodide molecules adsorbed at submonolayer coverage on the gold surface, which did not photodissociate, methyl bromide adsorbed on the same substrate at submonolayer coverage was easily photodissociated. Figure 6a displays a mass spectrum recorded from submonolayer (0.25 ML) CH_3_Br adsorbed on 10 ML Au/Mo(100). The spectrum was recorded at 90 K. The pump and probe wavelengths were 266 nm and 333 nm, respectively. The pump–probe delay time was fixed to 140 fs. As can be seen, the only observed photoreaction product was CH_3_^+^. Mass spectra recorded under different experimental conditions, i.e., different pump–probe delay times, laser intensities, coverages, and temperatures, did not lead to the detection of other reaction products.

The time evolution of this methyl cation signal is shown in Figure 6b. Similar to the real-time photodissociation of CD_3_I molecules on I/Au/Mo(100), which was discussed above, the CH_3_^+^ transient signal in Figure 6b exhibits a peak structure. The signal starts at 0 fs and no CH_3_^+^ transient signal was detected for reaction times longer than 600 fs. The measured data were also fitted with an exponential “rise and decay” model. The determined rise and decay time constants were τ_1_ = 60 ± 10 fs and τ_2_ = 90 ± 20 fs, respectively. The maximum of the peak structure was located at 140 ± 20 fs. The error of ±20 fs, in determining the maximum intensity, was derived from several transients recorded under similar conditions, i.e., similar substrate preparation, molecular coverage, and laser parameters. The other errors in determining the time constants were acquired directly from the fitting procedure.

**Figure 7:**
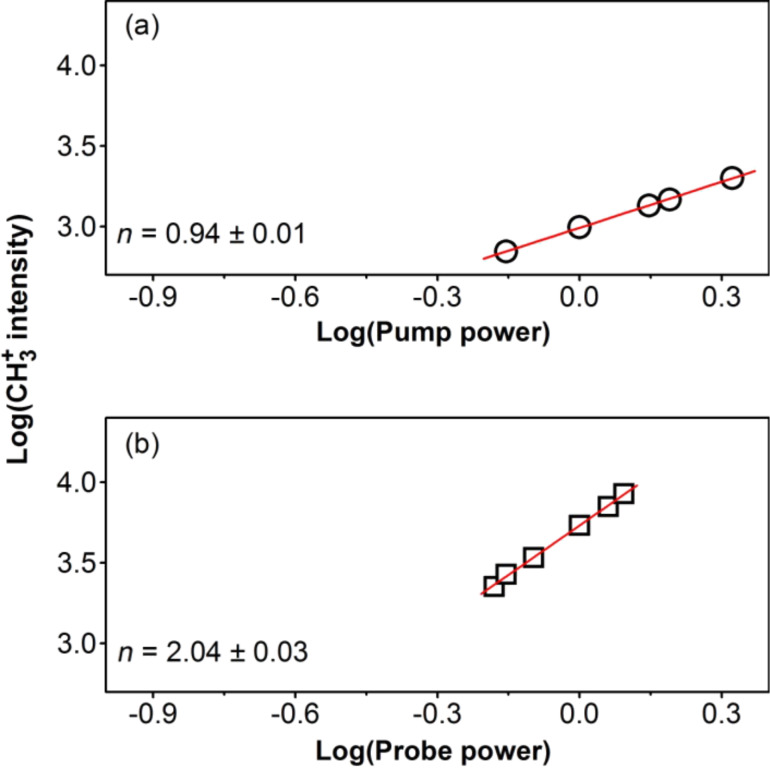
Intensity of the CH_3_^+^ signal as a function of (a) pump power and (b) probe power. Both measurements (a) and (b) were performed for a pump–probe delay time of 140 fs. The straight lines represent linear fits to the experimental data yielding the indicated slopes *n*.

Laser power dependence measurements indicated that the fundamental mechanism of the CH_3_Br photodissociation on the gold film is similar to that of methyl iodide on I/Au/Mo(100). The pump and probe power dependences of the CH_3_^+^ signal intensity at 140 fs pump–probe delay time are depicted in Figure 7 in a double-logarithmic representation. The slope of the graphs is an indication of the number of photons involved in the respective processes. From Figure 7 it can be seen that one pump photon was needed to excite the adsorbed CH_3_Br molecules. Therefore, the electron attachment mechanism might be possible, however, the two-photon probe power dependence renders this option again unlikely because rather three photons of 333 nm would be expected to be required to excite CH_3_Br^−^ to CH_3_Br^+^. The A-band of the free methyl bromide molecule has its maximum absorption cross section at around 6.2 eV [3]. The observation that CH_3_Br adsorbed on Au can be excited by means of a single photon with a central wavelength of 266 nm (4.7 eV) to a dissociative state, which is most likely the A-band, thus strongly indicates that the gold substrate induces a red-shift of the CH_3_Br A-band of about 1.5 eV. This energetic down-shift of the A-band states due to the interaction of the molecules with the gold surface is schematically indicated by the red dashed lines in Figure 2. Similar results were reported in the literature for CH_3_Br adsorbed on Ag(111) [34]. Figure 7b displays the quadratic power dependence measured for the probe laser beam, which confirms that two probe photons are most likely required to generate the ionized methyl fragments. Therefore, it is assumed that the excited adsorbate molecules were ionized by two nonresonant photons of 333 nm wavelength, and the ionized excited transition state decomposed yielding the CH_3_^+^ fragment.

The maximum intensity of the CH_3_^+^ transient peak obtained from the CH_3_Br photodissociation on gold is located at 140 fs (Figure 6b), whereas the transient peak of the CD_3_^+^ signal resulting from CD_3_I photodissociation on I/Au/Mo(100) was located at 50 fs with slightly different time constants for the rise and the decay of the signal. This is a clear indication of the different interaction of CH_3_Br with Au/Mo(100) compared to that of CD_3_I with I/Au/Mo(100). Thus, if the molecules dissociate by direct photoexcitation through the A state (which is proposed for methyl iodide and bromide), then these data represent intramolecular dynamics of the weakly perturbed molecule sitting on the Au surface, i.e., the dynamics in the transition-state region of photodissociation, which is intimately influenced by the detailed interaction with the surface. Clearly, further insight from first principles simulations on these systems would be highly desirable to complement the experimental data.

#### Methyl chloride

No light-induced formation of fragments was observed in the case of methyl chloride. This is most likely due to the fact that the A-band of CH_3_Cl with an absorption maximum at 7.3 eV above the electronic ground state of the free molecule [3] cannot be energetically lowered enough to become accessible to the 4.7 eV photons of the 266 nm pump laser beam.

## Conclusion

The present investigation demonstrates that time resolved pump–probe fs-laser time-of-flight mass spectrometry is able to probe the photodissociation dynamics of organic molecules on metallic substrates. The observed molecular dynamics varies strongly for the three investigated methyl halide molecules and reflects the different electronic structure of the molecules as well as the interaction with the gold substrate surface. The dissociation of methyl iodide on gold after UV irradiation was found to be quenched. In contrast, a modification of the gold substrate by slowly emerging iodine fragments was observed, which inhibited the quenching mechanism and enabled the measurement of transient methyl fragment signals. In the case of methyl bromide one-photon excitation was found to lead to the decomposition of the molecules on the gold surface. This was interpreted as an indication for a considerable red-shift of the A-band excitation of methyl bromide due to the interaction with the substrate. The alternative mechanism of dissociative electron attachment was found to be unlikely to be responsible for the observed dynamics of both molecules. Methyl chloride did not yield any detectable photofragments.

## Experimental

The experiments were performed in an ultrahigh vacuum (UHV) chamber equipped with standard tools for surface preparation and characterization [15,18,40]. Prior to deposition of the gold film, the surface was cleaned by heating the Mo(100) crystal to 2000 K by means of electron bombardment. Subsequently, the surface temperature was ramped down to 1000 K. In order to avoid the surface contamination, the Mo crystal was held at 1000 K by resistive heating until the background pressure restored (<5 × 10^−10^ mbar). Subsequently the surface was cooled down to the deposition temperature of 400 K. The gold evaporator was initially degassed and subsequently gave stable deposition rates over extended periods of time. In order to ensure the constant evaporation rate, prior to each deposition, the Au oven was preheated for 15 min. Subsequently, the substrate was positioned 10 mm away from the gold source and the support was held at 400 K. The gold evaporation was performed at normal incidence. The maximum background pressure in the UHV chamber reached throughout the gold evaporation process was less than 2 × 10^−9^ mbar. Deuterated methyl iodide was employed in the present study, but test measurements with CH_3_I yielded identical results. CD_3_I, CH_3_Br, and CH_3_Cl (all from Sigma-Aldrich, >99.5%, additionally purified by several freeze–pump–thaw cycles) were dosed onto the gold substrate at 90 K by means of an UHV compatible pulsed valve that was connected to a stainless steel tube ending close to the surface.

The fs pump (266 nm, third harmonic of fundamental, 80 fs, 1 mW/cm² average power, p-polarized) and probe (333 nm central wavelength, tuned by an OPA, 80 fs, 600 mW/cm² average power, p-polarized) were generated by a commercial amplified 1 kHz Ti:sapphire laser system (Spectra Physics Spitfire). The time zero in the experiments was determined in situ by monitoring the integral pump–probe time-dependent two-photon electron emission signal from the molybdenum surface.

Mass measurements were carried out on a homebuilt time-of-flight mass spectrometer [18,39]. The grounded Au/Mo(100) substrate served as the repeller electrode of a Wiley–McLaren-type acceleration lens arrangement [41]. The pump and probe laser beams collinearly irradiated the surface at an angle of 45°. Reaction products and intermediates that were ionized by the probe laser pulse were immediately removed from the surface and directed into the time-of-flight mass spectrometer by the static electric field between the substrate surface and the first acceleration electrode of the mass spectrometer. The ions pass a field free drift tube with different velocities according to their mass-to-charge ratio and are finally detected by a multichannel plate amplifier arrangement as a function of their flight time. To obtain the transient evolution of the product ion mass signals, the mass peaks were averaged over 2500 laser pulses for a fixed pump–probe delay time. The initial coverage was subsequently restored by admitting an identical amount of compound to the surface with the pulsed valve. Subsequently, the procedure was repeated for a new pump–probe delay time. Several of the thus obtained transients were averaged to yield the shown data.

## Supporting Information

Supporting information features the description of the characterization of the gold ultrathin films on Mo(100).

File 1 Characterization of ultrathin gold films on Mo(100).
