# Supplementary material for: Femtosecond time-resolved photodissociation dynamics of methyl halide molecules on ultrathin gold films
Source: Beilstein J Nanotechnol. 2011 Sep 20;2:618–27. doi: 10.3762/bjnano.2.65 (PMC3190631; doi:10.3762/bjnano.2.65)
Supplement: File 1 — Characterization of ultrathin gold films on Mo(100). [file Beilstein_J_Nanotechnol-02-618-s001.pdf]

Supporting Information

for

Femtosecond time-resolved photodissociation dynamics of methyl halide molecules on ultrathin gold films

Mihai E. Vaida, R. Tchitnga and Thorsten M. Bernhardt*

Address: Institute of Surface Chemistry and Catalysis, University of Ulm,

Albert-Einstein-Allee 47, 89069 Ulm, Germany

Email: Thorsten M. Bernhardt - thorsten.bernhardt@uni-ulm.de

* Corresponding author

Characterization of ultrathin gold films on Mo(100)

The gold deposition rate was calibrated by monitoring the intensities of the Auger transitions of Au N_7VV at 69 eV and Mo $M_5N_3N_5$ at 186.5 eV during direct evaporation of Au onto Mo(100) for different deposition times. Figure S1 displays an Auger electron spectrum recorded from 2 monolayers (ML) of Au evaporated onto Mo(100). As reference, an Auger electron spectrum recorded from a clean Mo(100) surface is presented as well. After evaporation of 2 ML gold, the intensity of the molybdenum MNN Auger transitions have slightly decreased, while the new peak at 69 eV becomes dominant. The inset in Figure S1 shows the Au to Mg Auger intensity ratio as a function of Au deposition time. A gradual increase of the gold coverage on the molybdenum surface led to a brake-point in the intensity ratio of the Au (69 eV) to Mo (186.5 eV) Auger transitions, which coincided with the exact amount of gold needed to complete the first Au layer on Mo(100). The brake point in the Au/Mo AES ratio appeared after a deposition time of 80 min indicating a deposition rate of 0.0125 ML/min.

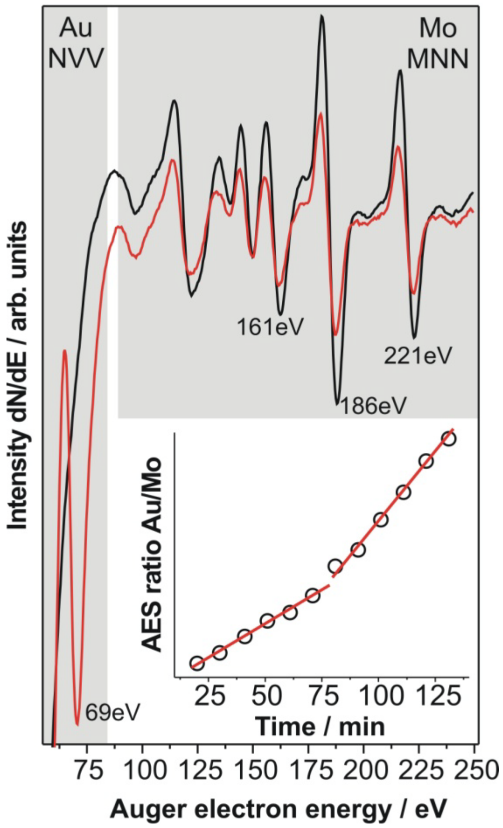

Figure S1: Auger electron spectra recorded from clean Mo(100) surface (black curve) and from a 2 ML Au film on Mo(100) (red curve). The inset shows the Au(N_7VV) to Mo($M_5N_3N_5$) Auger peak intensity ratio as a function of Au deposition time. The break point in the intensity ratio plot corresponds to the initial appearance of Au multilayers.

The electronic structure of the Au overlayer was also analyzed by means of fs-laser photoemission spectroscopy. The photoemission spectra measured in this laboratory have been reported previously [1,2] and exhibit similar features as the two photon photoemission spectra that have been obtained by Cao et al. from an Au(111) surface [3]. The work function deduced from the photoemission spectra was 5.02 ± 0.2 eV at an excitation wavelengths of 263 nm. This values is within 0.2 eV of that previously published by Hansson et al. (5.22 ± 0.04 eV) [4].

The structure of gold films consisting of 1 ML and of 10 ML Au on Mo(100) were characterized by means of low energy electron diffraction (LEED). Depending on the growth parameters, i.e., the deposition rate, the deposition temperature, and the annealing temperature, gold films with different structure can be obtained on

Mo(100). A strong dependence of the thin film structure on the growth parameters was reported for several transition metals deposited on single crystal refractory metal substrates, e.g., Pd/Nb(110) [5,6], Pd/Ta(110) [6,7], Pd/Mo(110) [8], Pd/W(110) [9], Cu/W(100) [10,11], Ag/W(100) [10-12], Ag/Mo(100) [10,11], Au/W(100) [11], Au/Mo(100)[11], Au/Mo(110)[13]. Only for Pd, Cu, Ag, and Au adsorbed at *sub-monolayer* coverage on Mo(100) comparable structural features were observed [11,14,15]. At *multilayer* coverage, however, the surface structure of these films depends strongly on the growth parameters as for all other systems mentioned above.

In the present work, gold was evaporated with a rate of 0.0125 ML/min on the Mo(100) substrate held at 400 K. Figure S2a–c shows LEED images recorded at 90 K from 1 ML gold coverage at different annealing temperatures. As can be seen, the freshly prepared surface (Figure S2a) appears amorphous because just a pale spot originating from the 00 reflex appears in LEED image. After 1 min annealing time at 800 K, the diffraction pattern corresponding to the (1×1) Mo(100) structure can be observed in Figure S2b.

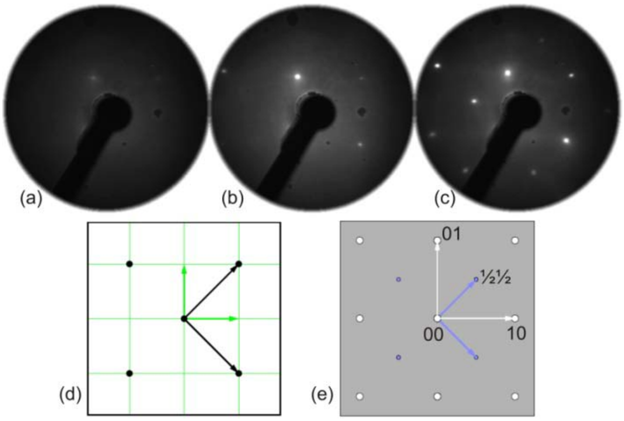

Figure S2: LEED images recorded from: (a) 1 ML Au on Mo(100); (b) 1 ML Au on Mo(100) annealed at 800 K for 1 min; (c) 1 ML Au on Mo(100) annealed at 1000 K for 1 min. The LEED patterns were recorded with a primary electron kinetic energy of E_P = 87 eV. (d) and (e) display a simulation of the c(2×2) Au/Mo(100) structure: (d) real lattice: the black points represent the Au atoms with respect the Mo substrate (thin green squares); (e) reciprocal lattice: the substrate spots are represented by the open circles while the Au spots are represented by the filled circles.

Further annealing at 1000 K for 10 min leads to the appearance of a c(2×2) structure in the LEED image as can be seen in Figure S2c for the 1 ML Au on Mo(100) preparation. A simulation of the c(2×2) LEED pattern employing the software LEEDpat 2.26 [16] is shown in Figure S2d and e. A similar c(2×2) structure was reported for Cu and Ag adsorbed at submonolayer coverage on Mo(100) as well, and it was in this case attributed to a surface rearrangement [10,17]. Subsequent investigations showed that the c(2×2) structure appears also for 0.5 ML Au/Mo(100) and it was attributed to the formation of a surface alloy in this case [11,15]. First principle calculations demonstrated that a c(2×2) substitutional surface alloy is energetically favored compared to overlayer structures [14]. Charge transfer from the Mo substrate to the adatoms is responsible for stabilizing the c(2×2) vacancy array, in which the adatoms are incorporated. Furthermore, the atomic radius of Au (1.44Å) is comparable with the atomic radius of Mo (1.39Å), which allows Au atoms to insert within the surface layer and to form a planar alloy structure [11].

A further increase in the Au coverage on the Mo(100) substrate induces another structural change of the surface. Figure S3a,b shows the diffraction patterns obtained from a 10 ML Au film on Mo(100). The freshly prepared surface appears amorphous, similar to the 1 ML preparation (cf. Figure S2a). After 1 min annealing at 1000 K, a new structure appears in the LEED image, which becomes more apparent when the annealing time is increased to 10 min (Figure S3b). In Figure S3c and d a LEEDpat simulation of a c(2×8) Au/Mo(100) structure is shown. The simulated c(2×8) structure resembles the marked structure presented in Figure S3b (see also Figure S3e). LEED and low energy electron microscopy investigations demonstrated for the case of Pd adatoms adsorbed on the Mo(100) that a change from a c(2×2) to a c(2×8) structure occurs by increasing the Pd coverage above 0.7 ML [14]. The c(2×8) structures was observed just for a deposition rate below 0.033 ML/min at substrate

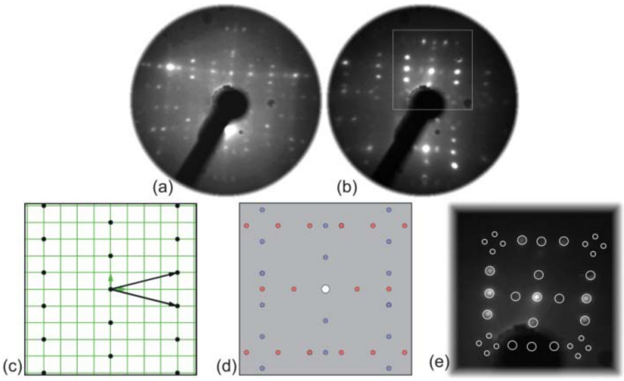

Figure S3: LEED images recorded from: (a) 10 ML Au on Mo(100), E_P = 87 eV; (b) 10 ML Au on Mo(100) annealed at 1000 K for 10min, E_P = 30 eV. (c) to (e) Simulation of a c(8×2) Au structure: (c) Real lattice: the black points represent the Au atoms with respect to the Mo(100) substrate (thin green squares); (d) Reciprocal lattice: the 00 reflex is represented by the open circle while the Au domain spots are represented by the filled circles. (e) Enlarged view into the domain structure marked in (b). For the sake of clarity the diffraction spots were highlighted with open circles.

temperatures larger than 800 K, whereas at higher deposition rates the surface structure was significantly different. The Pd/Mo(100) c(2×8) structure persisted at a coverage of 2.5 ML. The surface structure at coverages higher than 2.5 ML was not reported.

A detailed analysis of the LEED pattern obtained in the present work reveals a more complicated structure. Nevertheless, the obtained surface structure of the 10 ML Au film could be attributed to a degenerate c(2×8) structure comparable to the one observed Wu et al. [14]. The LEED patterns presented in Figure S3 are perfectly reproducible and are not influenced by a further thermal treatment of the surface, i.e. a higher preparation temperature or a longer annealing time.

References

1. Vaida, M. E., Bernhardt, T. M. *Conference Proceedings PHYSICS CONFERENCE TIM-10;* Timisoara, Romania, November 25–27, 2010; Bunoiu, M.; Malaescu, I., Eds.; American Institute of Physics: Melville, NY, 2011.
2. Vaida, M. E.; Gleitsmann, T.; Tchitnga, R.; Bernhardt, T. M. *Phys. Status Solidi B* **2010,** *247,* 1139. doi:10.1002/pssb.200945518
3. Cao, J.; Gao, Y.; Elsayed-Ali, H. E.; Miller, R. J. D.; Mantell, D. A. *Phys. Rev. B* **1998,** *58,* 10948. doi:10.1103/PhysRevB.58.10948
4. Hansson, G. V.; Flodström, S. A. *Phys. Rev. B* **1978,** *18,* 1572. doi:10.1103/PhysRevB.18.1572
5. Sagurton, M.; Strongin, M.; Jona, F.; Colbert, J. *Phys. Rev. B* **1983,** *28,* 4075. doi:10.1103/PhysRevB.28.4075
6. Strongin, M.; El-Batanouny, M.; Pick, M. A. *Phys. Rev. B* **1980,** *22,* 3126. doi:10.1103/PhysRevB.22.3126
7. Koel, B. E.; Smith, R. J.; Berlowitz, P. J. *Surf. Sci.* **1990,** *231,* 325. doi:10.1016/0039-6028(90)90201-I
8. Park, C.; Bauer, E.; Poppa, H. *Surf. Sci.* **1985,** *154,* 371. doi:10.1016/0039-6028(85)90040-8
9. Berlowitz, P. J.; Goodman, D. W. *Langmuir* **1988,** *4,* 1091. doi:10.1021/la00083a004
10. Soria, F.; Poppa, H. *J. Vac. Sci. Technol. (N. Y., NY, U. S.)* **1980,** *17,* 449. doi:10.1116/1.570479
11. Man, K. L.; Feng, Y. J.; Chan, C. T.; Altman, M. S. *Surf. Sci.* **2007,** *601,* L95. doi:10.1016/j.susc.2007.06.027

12. Attard, G. A.; King, D. A. *Surf. Sci.* **1989,** *222,* 360. doi:10.1016/0039-6028(89)90366-X
13. Pavlovska, A.; Paunov, M.; Bauer, E. *Thin Solid Films* **1985,** *126,* 129. doi:10.1016/0040-6090(85)90184-1
14. Wu, D.; Lau, W. K.; He, Z. Q.; Feng, Y. J.; Altman, M. S.; Chan, C. T. *Phys. Rev. B* **2000,** *62,* 8366. doi:10.1103/PhysRevB.62.8366
15. Man, K. L.; Feng, Y. J.; Altman, M. S. *Phys. Rev. B* **2006,** *74,* 085420. doi:10.1103/PhysRevB.74.085420
16. LEEDpat 2.26 PC-based software tool to visualize and analyze LEED patterns of substrates and overlayers; Fritz-Haber-Institut: Berlin, 2009.
17. Cerdá, J. R.; Soria, F.; Palomares, F. J.; de Andres, P. L. *Surf. Sci.* **1992,** 269–270, 713. doi:10.1016/0039-6028(92)91338-C
